# Glutathione Contributes to Caloric Restriction-Triggered Shift in Taurine Homeostasis

**DOI:** 10.3390/nu17050777

**Published:** 2025-02-23

**Authors:** András Gregor, Manuel Malleier, Arturo Auñon-Lopez, Sandra Auernigg-Haselmaier, Jurgen König, Marc Pignitter, Kalina Duszka

**Affiliations:** 1 Department of Nutritional Sciences, University of Vienna, Josef-Holaubek-Platz 2, 1090 Vienna, Austria; andras.gregor@univie.ac.at (A.G.); a01449161@unet.univie.ac.at (M.M.); sandra.haselmaier@univie.ac.at (S.A.-H.); juergen.koenig@univie.ac.at (J.K.); 2 Institute of Physiological Chemistry, Faculty of Chemistry, University of Vienna, Josef-Holaubek-Platz 2, 1090 Vienna, Austria; arturo.aunon-lopez@univie.ac.at (A.A.-L.); marc.pignitter@univie.ac.at (M.P.); 3 Vienna Doctoral School in Chemistry (DoSChem), Faculty of Chemistry, University of Vienna, Währingerstraße 42, 1090 Vienna, Austria; 4 Institute of Animal Nutrition and Functional Plant Compounds, University of Veterinary Medicine, 1210 Vienna, Austria

**Keywords:** taurine, glutathione, caloric restriction, intestine, bile acids, liver

## Abstract

Background/Objectives: Previously, we found that caloric restriction (CR) in mice increases taurine levels by stimulating hepatic synthesis, secretion into the intestine and deconjugation of taurine-conjugated bile acids (BA). Subsequently, in the intestine, taurine conjugates various molecules, including glutathione (GSH). The current study explores the mechanisms behind forming taurine-GSH conjugate and its consequences for taurine, other taurine conjugates, and BA in order to improve understanding of their role in CR. Methods: The non-enzymatic conjugation of taurine and GSH was assessed and the uptake of taurine, GSH, and taurine-GSH was verified in five sections of the small intestine. Levels of taurine, gavaged ^13^C labeled taurine, taurine conjugates, taurine-GSH, and GSH were measured in various tissues of *ad libitum* and CR mice. Next, the taurine-related CR phenotype was challenged by applying the inhibitors of taurine transporter (SLC6A6) and GSH-S transferases (GST). Results: The CR-related increase in taurine in intestinal mucosa was accompanied by the uptake and distribution of taurine towards selected organs. A unique composition of taurine conjugates characterized each tissue. Although taurine-GSH conjugate could be formed in non-enzymatic reactions, GST activity contributed to taurine-related CR outcomes. Upon SLC6A6 and GST inhibition, the taurine-related parameters were affected mainly in the ileum rather than the liver. Meanwhile, BA levels were somewhat affected by GST inhibition in the ileum and in the liver by SLC6A6 inhibitor. Conclusions: The discovered CR phenotype involves a regulatory network that adjusts taurine and BA homeostasis. GSH supports these processes by conjugating taurine, impacting taurine uptake from the intestine and its availability to form other types of conjugates.

## 1. Introduction

Caloric restriction (CR) is among the most effective interventions known to extend lifespan and enhance health outcomes across species [1]. Although the mechanisms behind these benefits remain not fully understood, emerging evidence indicates that metabolic adaptations involving key molecules such as taurine and glutathione (GSH) may play vital roles [2,3,4]. Understanding these adaptations could potentially lead to targeted therapeutic strategies that replicate CR’s benefits without the need for dietary restriction.

Taurine is one of the most abundant free amino acids in the body of mammals, particularly present in bile, intestine, heart, skeletal muscle, brain, nerve, liver, kidney, retina, and leukocytes [5]. The physiological requirements for taurine in mammals are supplied by dietary sources as well as by biosynthesis. Taurine can be found in various natural food sources as well as in infant formula, multiple energy drinks, and supplements. Within the body, ingested taurine is transported via the taurine transporter (SLC6A6) out of the intestinal epithelial cells, further carried with the blood, and absorbed into cells by SLC6A6 expressed in the destination tissues. Whereas taurine biosynthesis occurs mainly in the liver, brain, and kidneys, the production as well as turnover rate varies in different animal species [6,7]. Sulfur amino acids such as methionine and cysteine, as well as the enzymes cysteine dioxygenase (CDO) and cysteine sulfinate decarboxylase (CSAD), are required for taurine biosynthesis in the cysteine sulfinic acid or transsulfuration pathway [7]. The availability of sulfur amino acids regulates catalytic efficiency and degradation of CDO [8]. Taurine has anti-inflammatory, anti-oxidative, and osmoprotective properties. It plays a crucial role in reproduction and development and protects against various diseases and disorders [9,10,11,12].

As we showed previously, CR increases the concentration of taurine-conjugated BA in the liver of mice [13]. Upon secretion of BAs into the intestine, gut microbiota deconjugates BA and taurine. The released taurine forms various non-BA conjugates, which can be detected in the mucosa along the small intestine, in the plasma, and the liver [4,14,15,16]. Notably, the majority of the taurine conjugates, and thus, their potential role, still need to be characterized. So far, we have identified only taurine-chloramine (Tau-Cl) and taurine with GSH [4]. GSH is one of the most important non-enzymatic antioxidants, which serves as a scavenger of free radicals, aids in reducing H_2_O_2_, and takes part in detoxification. The occurrence of taurine-GSH conjugate in the intestine mucosa of mice is accompanied by an elevated expression and activity of GSH S-transferase (GST) [4]. GSTs are a family of enzymes that catalyze the conjugation of GSH to various electrophilic substrates. Interestingly, with selected molecules, GSH can create conjugates in non-enzymatic reactions [17,18,19]. The generated water-soluble conjugates are further directed to excretion via urine and bile [17,18,19]. Together with GSH peroxidases and GSTs, GSH forms the glutathione system, which is abundant in the gastrointestinal (GI) tract and relevant for maintaining gut health [20,21]. An impairment in intestinal GSH synthesis accompanies the development of inflammatory bowel disease (IBD) [22] and depletion of GSH in colon tissue occurs in patients suffering from chronic inflammatory disorders [23,24]. Accordingly, supplementation of GSH prevents lipid peroxidation and mucosal tissue damage in the rat model of colitis [25]. Similarly, providing *N*-acetylcysteine (NAC), which serves as a GSH precursor, reduces gut mucosa damage [26,27]. Notably, in our experiments, GSH, in conjunction with CR, enhances taurine uptake in the intestine [4].

In summary, we discovered a complex CR-related phenotype based on the tight connection between taurine and BAs. This regulation also has consequences for GSH and other molecules that form conjugates with taurine. However, the nature of the interaction of taurine with various molecules, as well as their function, remains unexplored. Similarly, unknown are the consequences of these interactions locally in the intestine and for other organs, particularly the liver, the main producer of taurine and BAs. As a gateway into the explored phenotype, we decided to focus on taurine-GSH, one of the very few taurine conjugates identified thus far. First, we aimed to investigate the nature of the conjugation reaction and its consequences. Further, we aimed to assess the occurrence of taurine-GSH and other conjugates in various organs and compare their distribution. Finally, knowing that GSH conjugation supports CR-triggered increase in taurine uptake, we challenged the CR phenotype by applying inhibitors of SLC6A6 and GSTs in *ad libitum*-fed and CR mice. We assessed the impact of the inhibitors on taurine, taurine conjugates, and BAs homeostasis in the intestine and liver.

## 2. Materials and Methods

### 2.1. Animal Care and Experimental Procedures

Male C57Bl/6 mice (Janvier Labs Le, Genest-Saint-Isle, France) were housed with a 12-h light/12-h dark cycle in standard specific-pathogen-free conditions. The animals were housed with free water access and fed a V153× R/M-H auto diet (SSNIFF-Spezialdiäten GmbH, Soest, Germany). The chow was composed of a blend of grains, minerals, and vitamins with no supplemented or restricted amount of taurine. At the age of 10 weeks, the animals were randomly divided into experimental groups of eight and underwent 14 days of ~20% calorie reduction or fed *ad libitum* as controls. The experimental setup is presented in the Supplementary Figure S1A. The CR mice were fed one time per day, with the defined amount of chow calculated based on average food intake during the week preceding the experiment. CR resulted in ca. 20% lower body weight compared to *ad libitum*-fed mice (Supplementary Figure S1B), which corresponded to comparable diet intake per gram body in CR and *ad libitum* at the end of the experiment. The sample size was calculated based on the differences in taurine concentration between *ad libitum*-fed and CR mice in previous experiments [10].

To trace taurine transport from the intestine, 2.7 mg of taurine-^13^C_2_ (Sigma, St. Louis, MO, USA) dissolved in water was gavaged to a group of five *ad libitum* and five 14-day CR animals. To prevent feed intake, the chow was removed from the cages three hours prior to the gavage. The animal organs were collected 2 h after the gavage.

In a separate experiment, the GST inhibitor ethacrynic acid (EA) [28] was administered to eight *ad libitum* and eight CR mice via intragastric bolus of 10 mg/kg every second day of the 14-day experiment.

Another group of eight *ad libitum* and eight CR animals received 1 mM imidazole-4-acetate (IAA) in drinking water over the 14 days of the experiment to inhibit SLC6A6 [29].

Urine and fecal samples were collected in metabolic cages. Mice were euthanized via isoflurane (Dechra Pharmaceuticals plc, Northwich, UK) overdose and a cardiac puncture. During the dissection, organs (intestine and colon sections, liver, kidney, spleen, heart, gastrocnemius muscle, prefrontal cortex) were dissected, snap-frozen, and stored at −80 °C until use. Mucosa samples from the colon and sections of the small intestine were obtained by cutting the organ open, washing it with PBS, and gently scraping the top tissue layer with a blade.

### 2.2. Intestinal Sacs Assay

The freshly dissected small intestine from the control groups *ad libitum* and CR groups (*n* = 5 completed sacs) was flushed with phosphate-buffered saline (PBS) and cut into segments. One end was sealed and the second was tied around a blunt needle. After the intestine was filled with 200 µL of a solution containing taurine (25 mg/mL) and GSH (61.5 mg/mL) in PBS, the needle was removed, and the tissue was tightly closed to create a four cm-long sac. After checking for any leaks, the sac was incubated in a 37 °C water bath containing 10 mL of prewarmed Dulbecco’s Modified Eagle Medium (DMEM, Sigma-Aldrich, St. Louis, MO, USA). Samples of the medium were collected at 0 min, 30 min, 60 min, and 90 min time points to measure taurine transport.

### 2.3. GSH, Taurine and Taurine Conjugates Assays and Detection

GSH and taurine standards (both from Sigma-Aldrich, St. Louis, MO, USA) were prepared in 70% ethanol. The analytical protocol followed previously published methods [4,15]. In brief, 7–10 mg of sample was placed in Precellys tubes with 1.4 mm ceramic beads and nine times the volume of methanol at −20 °C was added. The samples underwent homogenization in a Precellys^®^24 Tissue Homogenizer (Bertin Instruments, Montigny-le-Bretonneux, France) twice for two 15-s sessions at 5000 rpm, followed by 30 s of vortexing and 10 min of shaking on a laboratory rocker. Afterward, samples were centrifuged for 10 min at 18,000× *g*. The supernatants were then transferred to new tubes, and the centrifugation step was repeated. The final supernatants were placed in HPLC vials in a thermostatic autosampler maintained at 4 °C.

For the samples from intestinal sac and taurine-GSH incubation assays, 60 µL of the collected samples was diluted with 600 µL ethanol, vortexed, incubated for 20 min at −20 °C, and centrifuged at 15,000× *g* for 15 min at 4 °C. The supernatant was dried in a SpeedVac concentrator for 45 min at 60 °C, dissolved in 70 µL ethanol, and transferred into HPLC vials.

For the next assay involving taurine and GSH incubation, separate reactions containing 0.625, 1.25, 2.5, 12.5, 25, or 50 µg/mL of taurine and corresponding increasing concentrations of GSH were incubated for 30 min. The collected samples were processed for LCMS analysis, following an identical protocol as for samples obtained in the sacs assay.

All samples were analyzed by LCMS in negative mode applying an LCMS-8040 Liquid Chromatograph Mass Spectrometer (Shimadzu Corporation, Kyoto, Japan) equipped with an Atlantis T3 3 μm column (2.1 × 150 mm, Waters, Milford, MA, USA) at a constant temperature of 40 °C. Eluent A consisted of 0.1% formic acid in water, and eluent B of 0.1% formic acid in acetonitrile. The initial concentration of eluent B was 5% then a gradient was used for 8 min in a range of 5–20%, and the concentration was suddenly reduced to 5%.

To evaluate taurine, taurine-GSH, and GSH levels in various organs, nine to ten samples from control *ad libitum* and control CR samples were used. To measure the levels of other taurine conjugates in the organs, six to eight replicates were used. For the assessment of taurine, taurine-GSH, and other taurine conjugates in the samples from EA and IAA treatment, all available samples (*n* = 7–8) were used.

### 2.4. Bile Acid Detection

BAs were measured as previously described [30,31,32]. In summary, intestinal mucosa and liver samples were homogenized in Precellys tubes with ceramic beads with nine times the volume of ice-cold 100% methanol. Following homogenization, the samples were shaken on ice for 10 min, vortexed, and centrifuged for 10 min at 12,000× *g* at 4 °C. The supernatants underwent a second centrifugation for 10 min at 12,000 g at 4 °C, and the resulting supernatants were placed into HPLC vials and stored at 4 °C until analysis. The measurement was conducted in positive mode applying an LCMS-8040 Liquid Chromatograph Mass Spectrometer (Shimadzu Corporation) paired with an Atlantis T3 3 μm column (2.1 × 150 mm, Waters, Milford). Solvent A comprised water with 0.1% formic acid and 20 mmol/L ammonium acetate, while solvent B consisted of acetonitrile/methanol (3/1, *v*/*v*) with 0.1% formic acid and 20 mmol/L ammonium acetate. The solvent gradient was started at 30% B for 5 min, increased to 100% B at 25 min, and remained at that level for 20 min. To re-equilibrate, the composition was returned to the original gradient of 30% B for 10 min.

### 2.5. Gene Expression

RNA was isolated from intestinal mucosa using the RNeasy mini kit (Qiagen, Hilden, Germany) according to the manufacturer’s manual. For reverse transcription, SuperScript^®^ II Reverse Transcriptase (InvitrogenTM, Life Technologies, Carlsbad, CA, USA) was used. The QuantStudioTM 6 Flex Real-Time PCR System, along with the SYBR Green PCR Master Mix (both from Applied Biosystems, Life Technologies, Carlsbad, CA, USA) were used for the quantitative real-time PCR (qRT-PCR) reactions. The Supplementary Table S1 lists the primer pairs used for each gene. Eef1a1 served as a reference gene. The results are presented as average ΔΔCt for each experimental group.

### 2.6. Enzymatic Activity of Glutathione-S Transferases

GST activity was assessed using commercial assay kits according to the manufacturer’s indications (Sigma, Cat # CS0410). Tissues were disrupted in 5× volume PBS and immediately used for the measurement.

### 2.7. Statistics

Heatmaps illustrating the levels of taurine conjugates in experimental groups were generated using Z-Scored data, depicting the relative deviation from the groups’ mean value, and visualized using the MATLAB R2018b extension COVAIN v2019.04. The groups were arranged based on hierarchical clustering.

For sets containing two groups, comparisons were made using a two-sided Student’s *t*-test with a *p*-value lower than 0.05 was considered statistically significant. To assess statistical differences in experiments involving more than two comparisons, two-way ANOVA with Bonferroni correction for multiple testing was applied. In these cases, the differences with *p* < 0.05 but higher than the threshold following correction for multiple testing were referred to as trends to acknowledge potentially valuable findings while adhering to the strict threshold.

## 3. Results

### 3.1. CR Modulates Taurine and Taurine-GSH Uptake from the Intestine and Distribution into Other Tissues

**Figure 1:**
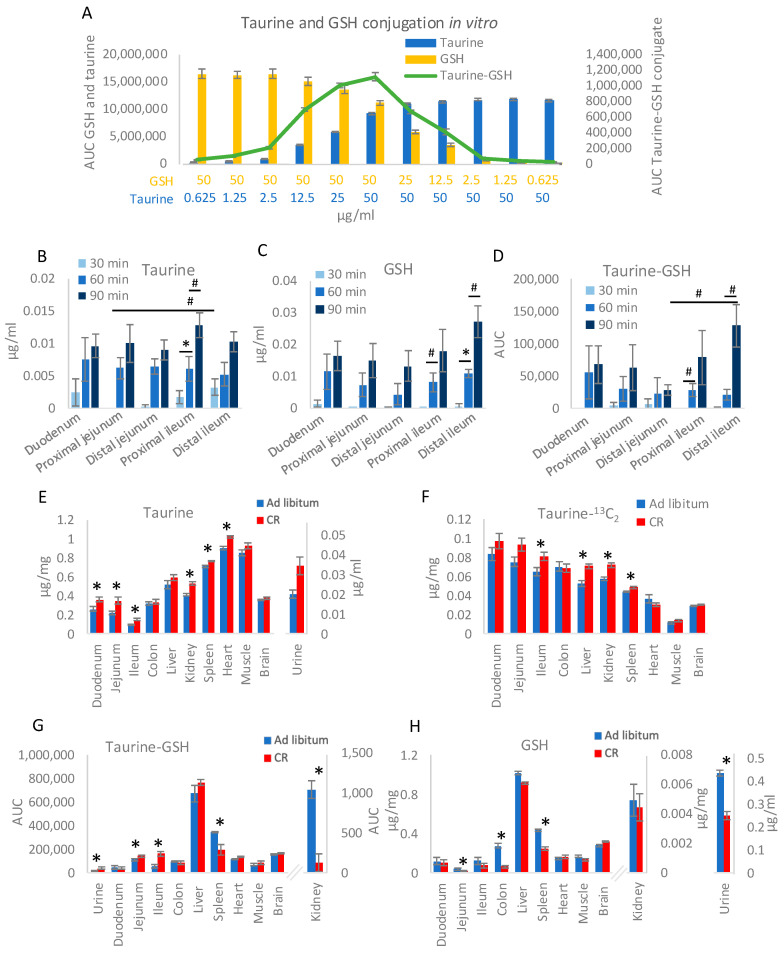
Caloric restriction (CR) modulates taurine uptake from the intestine and distribution into other tissues. Solutions containing taurine GSH at the indicated concentrations were incubated for 30 min and the levels of free taurine, taurine-GSH conjugate, and free GSH were assessed (**A**). Indicated sections of mice’s small intestine were filled with taurine and GSH solution and incubated at 37 °C in DMEM solution. The concentrations of taurine (**B**), GSH (**C**), and taurine-GSH conjugate (**D**) in the surrounding solution were measured in samples collected after 30, 60, and 90 min of incubation. Following two weeks of CR or control *ad libitum* feeding, the levels of free taurine (**E**), gavaged taurine-^13^C_2_ (**F**), taurine-glutathione (GSH) conjugate (**G**), and free GSH (**H**) were measured by applying HPLC-MS/MS in the indicated types of samples. The groups presented in panels (**B**–**D**) were compared using ANOVA and for these panels * represents statistical significance while # stands for a strong trend with *p* < 0.05 but is not statistically significant after correction for multiple testing. Two-tailed Student’s *t*-test was applied to assess statistical differences between the groups in panels (**E**–**H**) and * *p* < 0.05. Error bars indicate ±SEM. Figures in panels (**E**,**G**,**H**) represent the mean of nine to ten biological replicates. The data for panels (**B**–**D**,**F**) were prepared using five replicates and panel (**A**) using six.

The increased levels of taurine-GSH conjugate in the intestine of CR mice are accompanied by elevated activity of GSH S-transferases (GST) [4]. However, certain types of GSH conjugates can form in non-enzymatic reactions [17,18]. To assess whether taurine-GSH can be generated non-enzymatically, a range of taurine and GSH concentrations were incubated, followed by measuring the levels of free GSH, free taurine, and GSH-taurine conjugate in the solution. The formation of taurine-GSH depended on the concentration of both taurine and GSH and peaked at the highest µg/mL concentration of both compounds (Figure 1A).

The formation of taurine-GSH conjugate has been associated with a CR-triggered increase in taurine uptake from the ileum [4]. To investigate the patterns and potential connection between taurine and GSH uptake along the intestine, an ex vivo intestinal sacs assay was employed. Five sections of the small intestine were filled with taurine and GSH solution and the levels of taurine, GSH, and taurine-GSH were monitored in the medium surrounding the sacs over time. Already after 30 min, taurine could be measured in the medium containing duodenum, ileum, and, to a smaller extent, distal jejunum sacs (Figure 1B). However, the differences between the compartments of the small intestine for the 30-min time point were not statistically significant. Only the difference between proximal jejunum and distal ileum showed a strong trend (*p* = 0.03). In the proximal ileum samples, the increase in taurine concentration from 30- to 60-min time points was statistically significant, and a similar but non-statistically significant trend was measured for the difference between 60- and 90-min (*p* = 0.04). Comparable taurine concentrations were measured for all parts of the small intestine at the 60- and 90-min time points (Figure 1B).

Very small amounts of GSH were taken up from within the sacs during the first 30 min (Figure 1C). For 60- and 90-min time points, the changes in GSH concentration showed an interesting pattern with higher uptake in the duodenum, decrease in the jejunum, and peaking in the distal ileum (Figure 1C). Only the difference between 30- and 60-min time points for GSH conjugate in the distal ileum was statistically significant. The uptake of taurine-GSH conjugate along the small intestine followed a very similar pattern as GSH (Figure 1D). However, the difference in the uptake between the proximal and distal parts of the small intestine was more pronounced for taurine-GSH compared to GSH.

Knowing that taurine and GSH are taken up from the intestine and thus distributed with blood, the impact of CR on their levels in various tissues was assessed. First, we compared the levels of taurine in organs connected with the enterohepatic circulation of *ad libitum*-fed and CR animals: intestine (duodenum, jejunum, and ileum), proximal colon mucosa, and liver. Further, to assess the impact of CR-related taurine on other organs, we also analyzed the brain, spleen, heart, skeletal muscle, kidneys, and urine. In *ad libitum*-fed animals, taurine levels were generally higher in non-GI tissues, particularly the spleen, heart, and muscles, compared to the duodenum, jejunum, ileum, and colon (Figure 1E). Compared to Ad lib, CR triggered an increase in the levels of taurine in the mucosa of the duodenum, jejunum, and ileum, as well as urine, kidney, spleen, and heart (Figure 1E). No differences were detected in the liver (Figure 1E), confirming our previous report [4]. To verify if the difference in CR taurine levels in the kidney, spleen, and heart stem from increased local synthesis or transport from the intestine, Ad lib and CR mice were gavaged with taurine-^13^C_2_, and their organs were collected after 2 h. Increased levels of labeled taurine were found in the ileum mucosa, liver, kidney, and spleen, indicating enhanced intestinal uptake and transport of taurine to selected peripheral tissues in CR mice (Figure 1F).

CR is accompanied by the occurrence of multiple taurine conjugates in the intestinal mucosa, including taurine-GSH, which results in a reduction in the concentration of free GSH [4]. Correspondingly, in the current study, changes in taurine-GSH conjugate levels in the kidneys, urine, and spleen of CR mice were observed (Figure 1G). Whereas the concentration of free GSH decreased in the jejunum, colon, urine, and spleen of CR mice (Figure 1H). Corresponding with the lack of changes in taurine, taurine-GSH, and free GSH in the liver, there were no differences in hepatic GST activity (Supplementary Figure S1C). Notably, the changes in the levels of taurine-GSH conjugate in various tissues (Figure 1C) followed the pattern of GSH (Figure 1D) and not taurine concentration (Figure 1A).

### 3.2. CR Modulates Levels of Taurine Conjugates in Various Tissues

**Figure 2:**
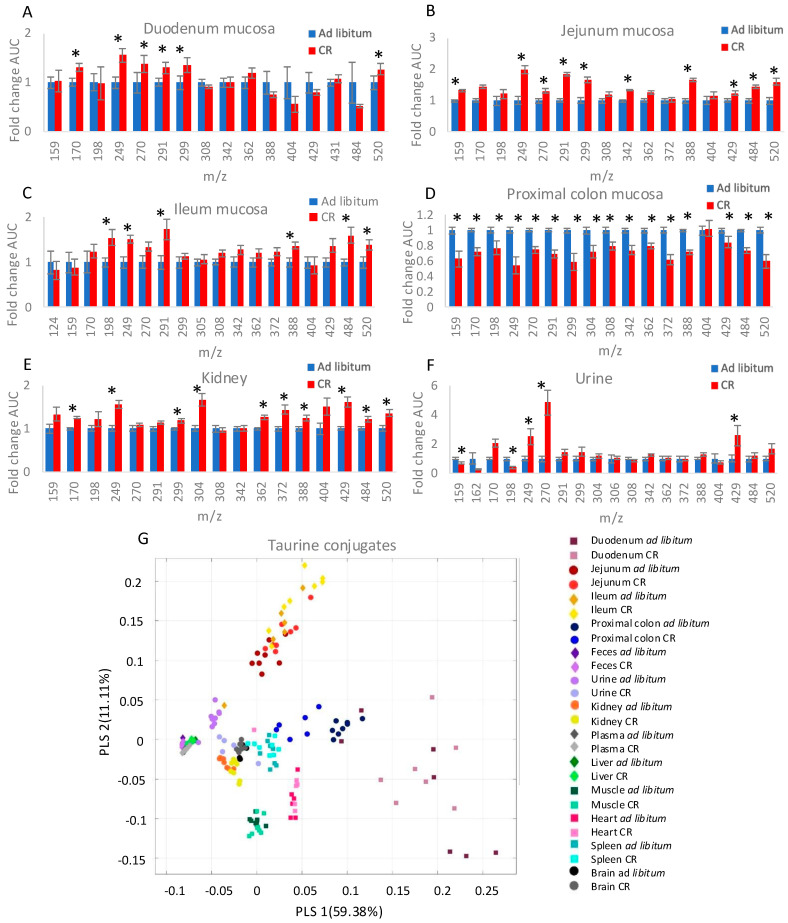
CR modulates levels of taurine conjugates in various tissues. The levels of taurine conjugates were measured in the mucosa obtained by scraping the top layer of the duodenum (**A**), jejunum (**B**), ileum (**C**), and proximal colon (**D**). Additionally, a fragment of the kidney obtained from the cross-section of the whole organ (**E**) as well as urine (**F**) were used for the measurement of taurine conjugate levels. The data was collected as an area under the curve of HPLC chromatogram peaks and presented as fold change. The regression of taurine conjugates in the listed tissues is based on covariance and depicted by a partial least square (PLS) chart (**G**). Statistical significance was assessed using a two-tailed Student’s *t*-test; * *p* < 0.05; *n* = 6–8. Error bars stand for ±SEM.

Besides taurine-GSH, taurine creates multiple other conjugates with unknown roles [4]. Seeing that taurine-GSH is present in various tissues, we decided to verify the presence and abundance of other taurine conjugates in the collected samples. We measured an increase in taurine conjugates in the mucosa of CR mice’s duodenum, jejunum, and ileum, with jejunum showing the highest number of statistically significant differences (Figure 2A–C). Importantly, the conjugate levels were reduced in the CR mucosa of the proximal colon (Figure 2D) and feces (Supplementary Figure S1D), confirming that the enhanced absorption of taurine in the small intestine results in its reduced levels in the distal GI tract. Interestingly, the levels of several taurine conjugates were higher in the kidney and urine of CR compared to *ad libitum*-fed mice (Figure 2E,F).

In the liver, neither taurine (Figure 1A) nor its conjugate levels were affected by CR (Supplementary Figure S1E). Interestingly, despite the increased levels of taurine in the spleen (Figure 1A), the only statistically significant changes in taurine conjugate levels concerned a decrease in CR mice, suggesting tissue-specific regulations in the ratio of free to conjugated taurine (Supplementary Figure S1F). Similarly, in the heart, where free taurine levels were increased, conjugates were not affected (Supplementary Figure S1G). The levels of three types of taurine conjugates were statistically significantly increased in CR mice plasma (Supplementary Figure S1H). Only minor changes in taurine conjugates composition were measured in the brain and skeletal muscles (Supplementary Figure S1I,J).

Besides taurine-GSH, the only other identified taurine conjugate was taurine-chloride (Tau-Cl, *m*/*z* 159). Its levels were increased in the mucosa of the jejunum and reduced in the mucosa of the proximal colon and urine (Figure 2A,B,D,F).

When comparing the occurrence patterns of various taurine conjugates in *ad libitum* and CR tissues, the sample clustering was strongly impacted by the type of tissue than the *ad libitum* and CR dietary conditions (Figure 2G). Interestingly, the heart and skeletal muscles clustered relatively close to each other (Figure 2G). Similarly, feces, plasma, and liver strongly overlapped (Figure 2G), even though the CR-dependent changes in the conjugate levels showed distinct patterns for these samples (Supplementary Figure S1D,E,H), further confirming that, in terms of taurine conjugates occurrence, tissue type-specificity has a stronger impact than exposure to CR.

### 3.3. Inhibition of SLC6A6 and GSTs Affect Taurine Homeostasis in the Small Intestine

**Figure 3:**
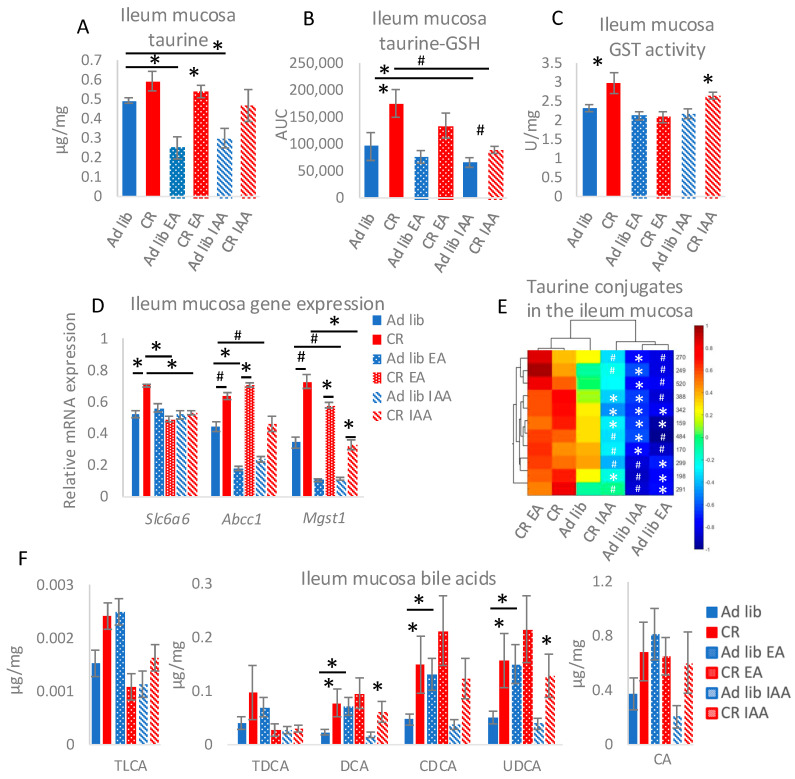
Inhibition of taurine transporter (SLC6A6) and GSH-S transferases (GST) affect taurine and bile acids (BA) homeostasis in the small intestine mucosa. In order to inhibit SLC6A6 and GSTs activity, mice submitted *ad libitum* and CR were given 1 mM imidazole-4-acetate (IAA) in drinking water or 10 mg/kg ethacrynic acid (EA) via intragastric bolus respectively. The levels of free taurine (**A**), taurine-GSH conjugate (**B**), other taurine conjugates (**E**), and BAs (**F**) were measured in the ileum mucosa applying HPLC-MS/MS. GST activity (**C**) and gene expression (**D**) were assessed in the mucosa of the ileum. Genes: *Abcc1*: ATP binding cassette subfamily C member 1 *Mgst1*: microsomal glutathione S-transferase 1; *Slc6a6*: solute carrier family 6 member 6; Bile acids: CA: cholic acid; CDCA: chenodeoxycholic acid; DCA: deoxycholic acid; LCA: lithocholic acid; TCA: taurocholic acid; TDCA: taurodeoxycholic acid; TLCA: taurolithocholic acid; UDCA ursodeoxycholic acid. ANOVA was applied to assess statistical differences between the groups. * represents statistical significance; # stands for a strong trend with *p* < 0.05 but is not statistically significant after correction for multiple testing; *n* = 8. Error bars represent ±SEM.

Seeing the interconnection between taurine and GSH in creating the conjugates and trafficking between tissues, we decided to disrupt taurine- and GSH-associated pathways to challenge their role in CR. For that purpose, *ad libitum*-fed and CR mice were given GST inhibitor EA or a competitive SLC6A6 inhibitor IAA. In order to verify the impact of EA and IAA on the uptake from the intestine, the taurine levels were assessed in the intestinal mucosa. As previously [4], CR tended to increase taurine concentration compared to *ad libitum* controls (Figure 3A). However, the increase was somewhat blunted in the IAA-treated groups (Figure 3A). Moreover, both *ad libitum* EA and *ad libitum* IAA groups differed statistically significantly from the control *ad libitum* group. Similarly, regardless of the treatment, the levels of taurine-GSH conjugate in the ileum mucosa maintained the pattern characteristic of *ad libitum*-CR differences (Figure 3B). However, IAA reduced the levels of taurine-GSH conjugate in *ad libitum* as well as CR group when compared to respective non-treated controls (Figure 3B). The CR-triggered increase in GST activity in the ileum mucosa was maintained in the CR IAA group but not in CR EA (Figure 3C). Notably, the EA did not inhibit GSTs’ activity below the level of the control *ad libitum* group (Figure 3C). The mRNA expression of Slc6a6, one of the GSH transporters ATP binding cassette subfamily C member 1 (Abcc1), as well as one of the GSTs microsomal glutathione S-transferase 1 (Mgst1), were affected by EA and IAA (Figure 3D). The strongest impact was measured for Slc6a6, where EA and IAA prevented the CR-specific increase in gene expression (Figure 3D). EA did not impact CR Abcc1 but reduced its expression in *ad libitum* mice (Figure 3D). Next, IAA partly reduced the impact of CR on Abcc1 mRNA levels and showed a tendency to reduce Abcc1 expression in *ad libitum* mice (Figure 3D). EA did not affect *Mgst1* gene expression compared to respective CR and *ad libitum* controls. Finally, IAA reduced the mRNA levels of Mgst1 in CR animals compared to CR control and showed a similar trend for *ad libitum* IAA compared to *ad libitum* controls. Despite that, the increase in CR IAA vs *ad libitum* IAA mice Mgst1 was statistically significant (Figure 3D).

To visualize the impact of the treatments on the levels of taurine conjugates, a heatmap was generated, which arranged the experimental groups according to the similarity of the conjugates profile (Figure 3E). For clarity, only the labels of statistical significance indicating the difference compared to the corresponding control *ad libitum* or CR group were marked in the heatmap. Correspondingly to taurine-GSH conjugate (Figure 3A), other taurine conjugate levels in the ileal mucosa were reduced by IAA in CR and *ad libitum* groups (Figure 3E). Notably, CR IAA conjugate levels were even lower than those of the *ad libitum* control group; however, they were still higher than those of *ad libitum* IAA (Figure 3E). The conjugates profile in the *ad libitum* EA group was very similar to that of the *ad libitum* IAA, and CR EA resembled CR control (Figure 3E).

As the rise in taurine concentration in the CR intestinal mucosa results from an increase in BAs concentration and deconjugation [4,13], the impact of EA and IAA on BAs was verified. EA partly disturbed CR-specific regulation of BA concentration by increasing the levels of several BAs, mainly in *ad libitum* mice (Figure 3F). Whereas IAA treatment did not differ from non-treated control, although the impact of CR was neutralized for taurine-conjugated BAs taurolithocholic acid (TLCA) and taurodeoxycholic acid (TDCA) (Figure 3F). Overall, EA showed a stronger impact on intestine BAs compared to IAA.

### 3.4. Inhibition of SLC6A6 Affects Hepatic Taurine and BA Homeostasis

**Figure 4:**
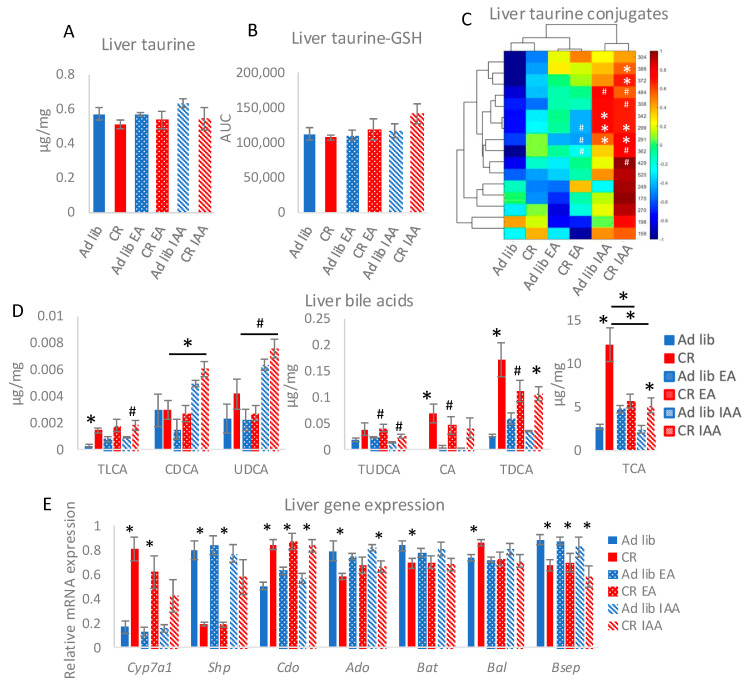
Inhibition of SLC6A6 affects hepatic taurine and BA homeostasis. The levels of free taurine (**A**), taurine-GSH conjugate (**B**), other taurine conjugates (**C**), and BAs (**D**) were measured in the liver. Gene expression (**E**) was assessed in the liver using qRT-PCR. Genes: *Ado*: 2-aminoethanethiol dioxygenase; *Bal*: bile acid CoA ligase; *Bat:* bile acid CoA:amino acid *N*-acyltransferase; *Bsep*: bile salt export pump; *Cdo:* cysteine dioxygenase; *Cyp7a1*: cholesterol 7-⍺-hydroxylase; *Shp*: small heterodimer partner. ANOVA was applied to assess statistical differences between the groups. * indicates statistical significance; # indicates a strong trend with *p* < 0.05 higher than the threshold set at 0.008, considering correction for multiple testing. Bars indicate the mean of seven to eight biological replicates ±SEM.

Since BAs and a substantial amount of taurine are synthesized in the liver and secreted in the GI tract, we assessed their levels in the liver of EA and IAA-treated animals. First, as previously reported [4], CR did not influence hepatic taurine or taurine conjugate levels (Figure 4A–C). Also, EA and IAA treatments did not affect hepatic levels of taurine and taurine-GSH conjugate (Figure 4A,B). Although, a minor, not statistically significant trend, was observed for an increase in taurine-GSH levels in CR IAA liver (Figure 4A,B). Contrary to the intestinal mucosa, hepatic taurine conjugate levels were increased by IAA, whereas EA did not affect it in the *ad libitum* group and had little impact on CR (Figure 4C).

Concerning hepatic BA, the differences between control *ad libitum* and CR were stronger than in the ileum mucosa and evident for taurine-conjugated BAs (TLCA, tauroursodeoxycholic acid (TUDCA), TDCA, and taurocholic acid (TCA)) (Figure 4D). EA and IAA decreased the levels of TCA in CR animals, but IAA increased or tended to increase chenodeoxycholic acid (CDCA) and ursodeoxycholic acid (UDCA) concentration in CR liver (Figure 4D). Overall, IAA showed a stronger impact on liver BAs compared to EA.

To verify the impact of the EA and IAA on the pathways connected with BAs and taurine, the expression of hepatic genes was quantified. Interestingly, we observed that IAA decreased the difference between *ad libitum* and CR mice in the expression of main factors regulating BA synthesis cholesterol 7-⍺-hydroxylase (Cyp7a1) and small heterodimer partner (Shp) (Figure 4E). CR increased the expression of Cdo but reduced Ado, both involved in the synthesis of taurine. EA neutralized the difference in 2-aminoethanethiol dioxygenase (Ado) expression between *ad libitum* and CR animals. Concerning genes connected with taurine conjugation, bile acid CoA:amino acid *N*-acyltransferase (Bat) levels were reduced by CR while bile acid CoA ligase (Bal) was stimulated, and EA and IAA neutralized the CR-triggered changes. Finally, the transporter’s bile salt export pump (Bsep) mRNA levels were reduced by CR, and this persisted regardless of EA and IAA treatment (Figure 4E).

## 4. Discussion

In summary, the study showed that although taurine-GSH conjugate can be generated in a non-enzymatic reaction, GST activity affects taurine uptake. Furthermore, CR triggers an increase in taurine levels in the small intestine and taurine transport to other organs. Next, the levels of taurine as well as taurine-GSH conjugates vary between different tissues. Similarly, we found that various taurine conjugates have distinct patterns of occurrence, which are influenced by the tissue type rather than CR feeding. Next, we show that both SLC6A6 and GSTs contribute to CR-related changes in taurine and GSH levels, which is further reflected in the levels of BAs and various taurine conjugates. Notably, like CR phenotype, the impact of SLC6A6 and GST inhibitors on taurine and its conjugates is predominantly observed in the intestine compared to the liver.

The presented data shows that the previously reported capacity of GSH to create conjugates spontaneously [17,18] applies also to taurine-GSH conjugate. Based on our observation, the in vitro conjugation rate increases linearly with the concentration of GSH and taurine. Importantly, the molar mass of GSH (307.33 g/mol) is higher than taurine (125.15 g/mol), thus the highest level of taurine-GSH conjugate at 50:50 ug/mL reflects approximately 1:2.5 ratio of GSH to taurine molecules. That is likely why, compared to other tested samples, the highest amount of taurine-GSH is in the liver, where also free taurine and GSH occur in relatively high concentrations. Likely, in the small intestine, where GSH concentration is lower, GSTs may support the production of the conjugates. Alternatively, GSTs may catalyze reactions competing with non-enzymatic reactions.

The fact that GSH plays a vital role in REDOX response suggests potential negative consequences of the CR-triggered reduction in free GSH levels. However, our previously reported data showed that while CR resulted in the increased expression and activity of GSTs accompanied by reduced GSH/GSSG ratio, it did not trigger changes in the expression of GSH synthesis (glutathione synthase, glutamate-cysteine ligase, gamma-glutamyl transpeptidase) and oxidation/reduction-related genes (glutathione reductases 1 and 2 as well as glutathione peroxidase 1 and 2) as well as no increase in the gene expression of other oxidative-stress responsive factors (manganese superoxide dismutase, catalase, thioredoxin 1, and 2) in the intestine mucosa. Moreover, levels of reactive oxygen species and anti-oxidative capacity remained unaffected in CR intestinal mucosa compared to *ad libitum* controls [4]. This may be explained by the fact that CR reduces oxidative stress by increasing mitochondrial efficiency while decreasing metabolic rate and inflammation [33,34,35,36]. Consequently, diminished oxidative stress may result in reduced requirements for GSH. Moreover, taurine-GSH conjugate is not permanent and may dissolve when one or both molecules are needed for their other activities. Finally, it is important to consider that taurine also displays antioxidative properties [37,38], and thus, it may support GSH’s protective function.

Regrettably, the physiological role of the majority of taurine conjugates is still not known. Since, so far, only a few examples of substances creating non-enzymatic conjugates with GSH are known [17,18], it is not clear which reactions are spontaneous and which potentially require enzymatic support. A possible option is that upon taurine surplus, taurine-GSH may serve as a storage and/or temporary deactivation of otherwise biologically functional molecule. Alternatively, the conjugate itself plays a biological role, e.g., support of taurine uptake in the intestine [4]. Importantly, there is no method that allows the deconjugation rate to be measured. Moreover, we neither know if deconjugation occurs spontaneously or if it depends on each molecule’s concentration, which could reflect taurine and GSH utilization in reactions with other molecules.

Using ex vivo sacs, we found that the transport of taurine and GSH varies somewhat along the small intestine. At the 30-min time point, taurine was already present in the external solution, while GSH was not yet. Both molecules were transported more efficiently in the distal part of the small intestine. Interestingly, the uptake of taurine-GSH conjugate followed a similar pattern as the uptake of GSH, but with bigger differences between the 60- and 90-min time points. SLC6A6 and ABCC1 serve as intestine transporters of free taurine and GSH, respectively [39]. Additionally, ABCC1 may transport GSH conjugates [39]. Thus, taurine-GSH may be taken up with the same transporter as free GSH, which would explain the similarities in the uptake pattern along the small intestine. Previously, we described that GSH enhances the efficiency of taurine uptake in CR animals and that Slc6a6 expression increases in CR intestine [4]. Notably, here we also show that the expression of Abcc1 increases in the ileum of CR mice.

As mentioned above, the rate of non-enzymatic conjugation and deconjugation of taurine and GSH is unknown, which hinders the interpretation of sac assay results. When measuring taurine-GSH in the solution surrounding the sac, the outcomes may result from the transport of the conjugate or taurine and GSH separately, and the conjugation may take place subsequently. Correspondingly, the assessed levels of free taurine and free GSH may reflect their uptake by the respective transporters or transfer of taurine-GSH conjugate, which is then deconjugated.

Knowing that (1) the liver increases BA synthesis and secretion during CR, (2) the elevated concentration of taurine in the GI tract stems from the deconjugation of BA, and (3) CR enhances the efficiency of intestinal uptake of taurine [4,15], we assumed that the increased level of taurine in tissues outside of enterohepatic organs in CR animals stems from the intestinal pool. Gavaging labeled taurine, we confirmed this hypothesis and proved that the taurine from the intestine is transported to other organs and that in the CR, compared to *ad libitum* mice, the liver, kidneys, and spleen take up increased levels of intestine-derived taurine. Thus, our results imply that the modulation of taurine biosynthesis in the CR liver influences taurine levels in specific organs besides the GI tract and that the transfer of hepatic taurine to other tissues occurs via secretion into the small in the form of taurine-conjugated BAs. The reason for enhanced hepatic synthesis of taurine during CR remains undetermined. One intriguing possibility is that since taurine stimulates the production of BA [40,41,42,43], this may be its primary function in CR liver, and its release in the intestine is only a derivative of this process. This hypothesis fits with the increased clearance of taurine as the side product of the physiological circumstances by secretion in CR mice urine.

Importantly, we measured an increase in labeled taurine levels in only several tissues of CR mice; however, other tissues also contained labeled taurine, but the amount did not differ between *ad libitum* and CR mice. This suggests that CR may selectively increase SLC6A6 expression and taurine availability in specific target tissues. However, it is not known why certain organs are more prone to taurine uptake during CR than others and what the potential role of taurine in various tissues during CR is.

Dietary taurine affects the levels of taurine throughout the body, and it has a particularly strong impact on the kidney and its function. The expression and activity of renal epithelial SLC6A6, which is involved in the reabsorption of taurine, is regulated by dietary sulfur amino acids; e.g., a low-taurine diet upregulates and taurine supplementation down-regulates SLC6A6 in kidneys [44,45,46,47]. Consequently, in animals fed a low-taurine diet, urinary taurine and fractional excretion of taurine are reduced compared to control, while taurine supplementation increases it [46,48]. We measured that the concentration of taurine in kidneys and secretion with urine is increased in CR animals compared to *ad libitum* mice. Importantly, CR is associated with a reduction of nutrient intake, including taurine. Thus, the secreted taurine stems from biosynthesis. However, it is important to consider that the difference in urine taurine levels between *ad libitum* and CR animals is much smaller than in the reported cases of taurine supplementation vs. non-supplemented controls. Additionally, the increased concentrations of free taurine in the urine of CR animals suggest that despite preventing the secretion of taurine and its conjugates with feces, part of it is removed in the urine.

Regarding taurine levels, we showed previously that the CR-stimulated taurine synthesis in the liver is reflected in an increase in free taurine concentration in the intestine but not in the liver. This stems from the fact that a substantial amount of hepatic taurine is stored in the form of conjugated BAs, the majority of which are is deconjugated upon secretion in the intestine [4,15,49]. Moreover, we showed that while CR influences taurine homeostasis in the intestine, other factors like macronutrient intake impact hepatic taurine and its conjugate levels [49]. Similarly, upon application of IAA and EA, the main differences in taurine and its conjugate levels were measured in the intestine, while in the liver, only several taurine conjugates were affected. This observation further hints that despite the dynamic exchange via enterohepatic circulation, the liver and intestine show distinct adaptations in terms of preferred ranges of taurine concentration. Moreover, it depicts buffering capacity on multiple levels, where the concentration of free taurine is modulated by dietary intake, biosynthesis, as well as conjugation to BA, GSH, and other compounds, depending on the type of organ. Finally, these results imply that in an attempt to estimate total taurine levels, the measurements need to take into account the physiological circumstances, the type of tissue, and the molecules taurine is capable of interacting with.

Overall, we observe that even though the conjugation of taurine and GSH may occur spontaneously, inhibition of GSTs affects the levels of taurine conjugates in the intestinal mucosa, confirming our previous report [4]. However, hepatic taurine and taurine conjugate levels are not affected by CR; thus, the inhibition of GSTs did not trigger changes in any of the parameters assessed in the liver in this study. Moreover, by measuring GSTs’ enzymatic activity, we could prove that EA treatment prevented CR-triggered increase in GSTs’ activity. Interestingly, it did not lower it below the *ad libitum* level.

In our previous publications, we showed that CR modulates the expression of hepatic genes connected with BA and taurine synthesis, conjugation, and transport, including stimulation of *Cyp7a1*, *Cyp27a1*, *Cdo*, *Bal*, and *Ntcp* and repression of *Shp*, *Bat*, and *Bsep* [4,13]. Here, we confirm previous results and additionally report CR-related reduction of *Ado* gene expression. Also, we found that disturbance in taurine transport affects the expression of the main factors responsible for BA synthesis, *Cyp7a1*, and *Shp*, which is reflected in reduced levels of the most abundant BA in the liver TCA. Upon secretion to the GI tract, most BAs are deconjugated, metabolized by gut microbiota, and reabsorbed. Thus, the hepatic and intestinal BA pools do not overlap, also in this study. Importantly, in both organs, CR triggers an increase in BA levels, as published previously [4,13]. Finally, EA and IAA showed slight differences in their impact on BA, with EA affecting more BAs in the ileum and IAA in the liver.

The functional purpose of the increased taurine concentration in the CR GI tract remains unknown. Considering the anti-inflammatory properties of taurine [9,50,51,52,53], also in the intestine [54], we could speculate that it may mediate part of the outcomes of CR. We have shown that CR results in the downregulation of the immune genes in the intestine of the healthy and DSS-colitis mouse model [16,55]. Importantly, taurine has also been shown to counteract colitis [12,56,57]. Furthermore, both taurine supplementation and CR enhance mitochondrial function expression and reduce endoplasmic reticulum and oxidative stress [58,59,60,61,62]. Also, both support body weight loss and the treatment of diabetes [63,64,65,66]. Importantly, CR and taurine are recognized for life-extending properties [61,67]. Interestingly, taurine deficiency has recently been recognized as a driver of aging, and the plasma concentration of taurine in mice, monkeys, and humans declines with age [68]. Based on our studies, CR may be a way to offset these changes. However, further research focusing on common CR- and taurine-affected molecular pathways, as well as potential novel applications of taurine, are needed.

## 5. Conclusions

In conclusion, we show that taurine creates various conjugates and their composition varies between tissues, suggesting involvement in distinct organ-specific processes. Moreover, the occurrence of specific types of conjugates is impacted stronger by the type of tissue than by *ad libitum* or CR feeding. Thus far, only two taurine conjugates have been identified as taurine-GSH and TauCl and their role is partly understood. Therefore, a range of taurine conjugates requires further investigation, with particular focus on their potential tissue-specific role.

Finally, taurine plays the central role in the CR phenotype we discovered. However, a complex network of interactions and physiological adjustments occurs as a response to CR, and GSH substantially supports the functionality of these regulations. In the following studies, the physiological consequences of the interaction between taurine and GSH, as well as their potential contribution to the beneficial outcomes of CR, need to be addressed.

## Data Availability

The raw data supporting the conclusions of this article will be made available by the authors on request.

## Supplementary Materials

The following supporting information can be downloaded at: https://www.mdpi.com/article/10.3390/nu17050777/s1, Figure S1: Graphical presentation of the experimental procedures (A). The body weight of the animals was measured at the end of the experiment (B). GSTs’ activity was measured in the liver using a commercial assay kit (C). The levels of taurine conjugates were measured in the feces (D), liver (E), spleen (F), heart (G), plasma (H), brain (I), and muscle (J) of ad libitum-fed and CR mice using HPLC-MS/MS. n = 6–8; * *p* < 0.05.; Table S1: Primers used for qRT-PCR.

## Abbreviations

ABCC1 ATP	binding cassette subfamily C member 1
ADO 2	aminoethanethiol dioxygenase
BAL	bile acid CoA ligase
BAT	bile acid CoA:amino acid *N*-acyltransferase
BSEP	bile salt export pump
CA	cholic acid
CDCA	chenodeoxycholic acid
CDO	cysteine dioxygenase
CSAD	cysteine sulfinate decarboxylase
CR	caloric restriction
CYP7A1	cholesterol 7-⍺-hydroxylase
DCA	deoxycholic acid
DMEM	dulbecco’s modified eagle medium
EA	ethacrynic acid
GI	gastrointestinal
GSH	glutathione
GST GSH-S	transferases
IAA	imidazole-4-acetate
LCA	lithocholic acid
MGST1	microsomal glutathione S-transferase 1
PBS	phosphate-buffered saline
SHP	small heterodimer partner
SLC6A6	solute carrier family 6 member 6
Tau-Cl	taurine-chloramine
TCA	taurocholic acid
TDCA	taurodeoxycholic acid
TLCA	taurolithocholic acid
TUDCA	tauroursodeoxycholic acid
UDCA	ursodeoxycholic acid
