# Supplementary material for: Glutathione Contributes to Caloric Restriction-Triggered Shift in Taurine Homeostasis
Source: Nutrients. 2025 Feb 23;17(5):777. doi: 10.3390/nu17050777 (PMC11901847; doi:10.3390/nu17050777)

Supplementary figures

Supplementary Table S1: Primers used for qRT-PCR.

Gene ID	*Forward*	*Reverse*
Ado	CCAGGACGTGTTTGGGATTGT	ACCAGTCTTGACACTGTAGTGA
Eef1a1	CCTGGCAAGCCCATGTGT	TCATGTCACGAACAGCAAAGC
Bal	TGTGTGTGAAGGAACCTGGA	ACCCGGACAACTTTGTGAAG
Bat	TAGAGCACACCACGTTCCTG	GCACAGGCTCATCAACAAGA
Bsep	AGACAGGCAACCCGTCATGGACT	ACGAACGCCGTCGTTTCCCC
Cdo	GGGGACGAAGTCAACGTGG	ACCCCAGCACAGAATCATCAG
Cyp7a1	TGTCTGCGAGGGCTGGAGCA	CCAGCCTGGGATGCTATGGGC
Mgst1	CCTTCTCCCTGGATTCAGTCAT	TCGGCCATGCTTCCAATCTT
Mrp1	CCTTCTCCCTGGATTCAGTCAT	TCGGCCATGCTTCCAATCTT
Shp	CTCATGGCCTCTACCCTCAA	GGTCACCTCAGCAAAAGCAT
TauT	GCACACGGCCTGAAGATGA	ATTTTTGTAGCAGAGGTACGGG

Supplementary Figure S1

Graphical presentation of the experimental procedures (A). The body weight of the animals was measured at the end of the experiment (B). GSTs' activity was measured in the liver using a commercial assay kit (C). The levels of taurine conjugates were measured in the feces (D), liver (E), spleen (F), heart (G), plasma (H), brain (I), and muscle (J) of *ad libitum*-fed and CR mice using HPLC-MS/MS. n=6-8; *p<0.05.

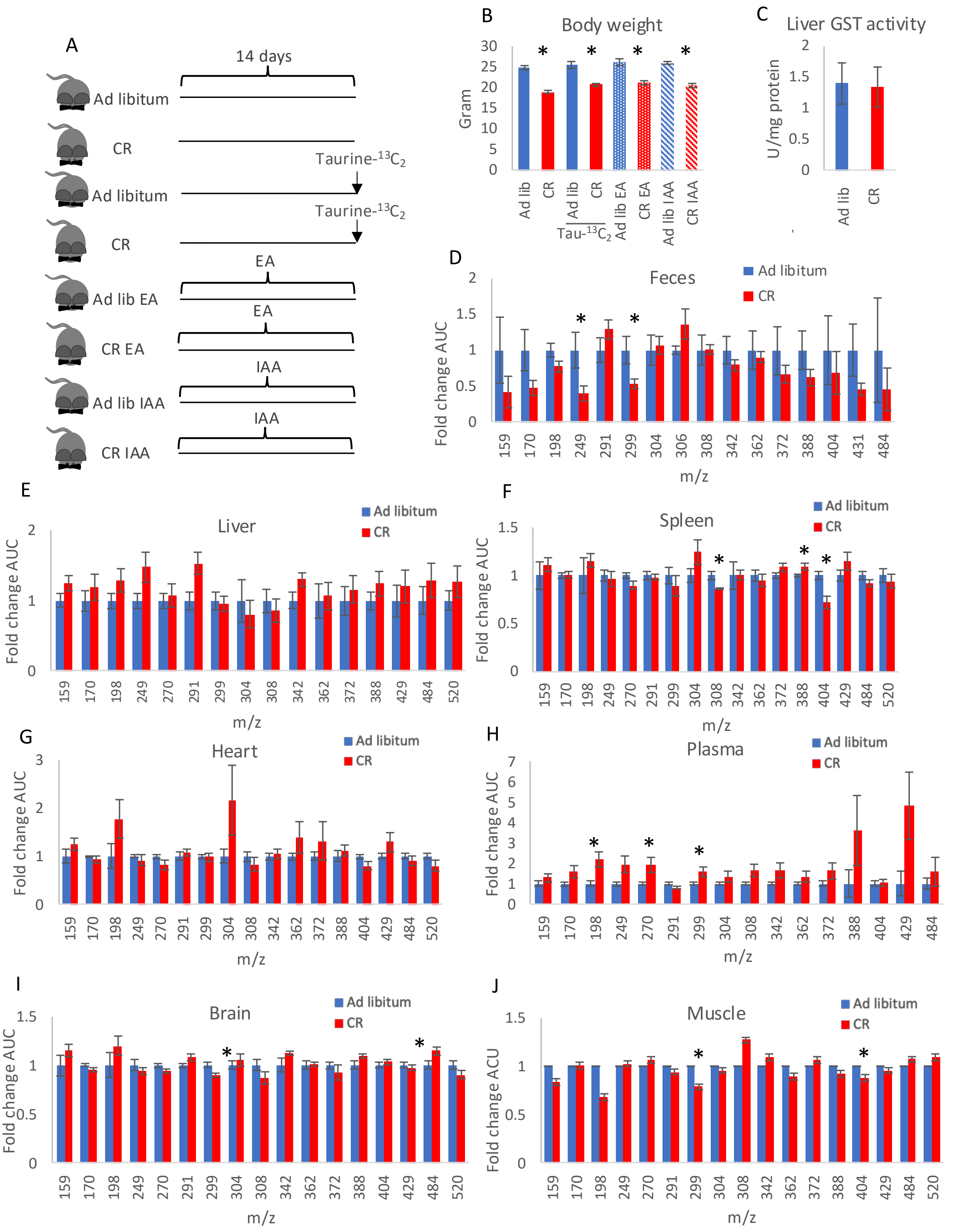

Supplement: Supplementary file 1 [file nutrients-17-00777-s001.zip › nutrients-3442716-supplementary.pdf]
